# The Interplay of Cumulative Perioperative Morbidity and Health-related Quality of Life After Radical Cystectomy—Prospective Evidence from the COMPACT Registry

**DOI:** 10.1016/j.euros.2025.09.013

**Published:** 2025-10-11

**Authors:** Mara Koelker, Sandra Trepte, Jakob Klemm, Markus von Deimling, Hanna Kukuk, Andreas Wirtz, Adrian Pachollek, Felix Luebbersmeyer, Tim A. Ludwig, Roland Dahlem, Margit Fisch, Malte W. Vetterlein

**Affiliations:** Department of Urology, University Medical Center Hamburg-Eppendorf, Hamburg, Germany

**Keywords:** Patient-reported outcome measures, Postoperative complications, Quality improvement, Quality of life, Urinary bladder neoplasms

## Abstract

**Background and objective:**

Radical cystectomy (RC) in bladder cancer patients is associated with considerable short-term morbidity. Although RC is known to impair health-related quality of life (HRQOL), the impact of complication severity on HRQOL in the long term remains unclear. The aim of this study is to investigate the relationship between perioperative cumulative complication burden and HRQOL after RC using data from a prospective registry, given the limited existing evidence.

**Methods:**

The Comprehensive Outcome Measures and Perioperative Morbidity After CystecTomy (COMPACT) registry (DRKS00024929) prospectively collects standardized data on perioperative morbidity and longitudinal patient-reported outcome measures. The study includes patients undergoing open RC with pelvic lymph node dissection and urinary diversion for bladder cancer. According to the European Association of Urology guidelines, 90-d morbidity was assessed using both the Clavien-Dindo classification (CDC) and the Comprehensive Complication Index (CCI). HRQOL was measured at baseline and 3, 6, and 12 mo using the Functional Assessment of Cancer Therapy—Bladder—Cystectomy (FACT-BL-CYS) scores (range 0–168). Patients treated between 2020 and 2022 were included. Multivariable linear regression was used to evaluate the associations of 90-d CDC grade ≥IIIb (ie, “major complications”) and 90-d CCI with the 6-mo FACT-BL-CYS total score, adjusting for clinical and pathological confounders.

**Key findings and limitations:**

Among 82 patients, one (1.2%) had no complications, and 47 (57%) had CDC grade ≤II, 22 (27%) grade III, and 11 (13%) grade IV complications. The 90-d mortality rate was 1.2%. The median 90-d CCI was 35 (interquartile range [IQR] 26–45). The median 6-mo FACT-BL-CYS total score was 119 (IQR 90–142). Only comorbidity (age-adjusted Charlson index) was significantly associated with HRQOL (coefficient: –4.76, *p* = 0.02); neither CDC ≥IIIb (*p* = 0.7) nor CCI (*p* = 0.2) was significant. Limitations include uncertainty in effect sizes due to the low number of major complications.

**Conclusions and clinical implications:**

Open RC is associated with a high rate of perioperative complications when assessed with standardized methods. However, our findings suggest that their impact on HRQOL at 6 mo is limited. HRQOL appears to be more closely related to age-adjusted comorbidity. These insights should inform preoperative counseling and guide individualized postoperative care planning.

**Patient summary:**

We looked at whether complications after bladder removal surgery (radical cystectomy) affect patients’ quality of life. We found that most patients have complications, but these usually do not reduce quality of life 6 mo after surgery. Instead, pre-existing health conditions had a stronger impact on recovery.

## Take Home Message

Despite the high rate of complications after radical cystectomy, these complications do not significantly affect quality of life at 6 mo postoperatively. Pre-existing comorbidity, rather than surgical morbidity, appears to be the key driver of patient-reported recovery.

## 1 Introduction

Radical cystectomy (RC) in bladder cancer patients is a major surgical procedure associated with considerable short-term morbidity [1]. To improve comparability of outcomes, the European Association of Urology (EAU) introduced a guideline for procedure-specific complication reporting [2], emphasizing the Clavien-Dindo classification (CDC) [3], which is used widely to grade the most severe complications [4]. However, the CDC suffers from inter-rater variability, and standardized definitions for RC-specific complications are lacking, limiting reproducibility and comparability [5]. To address these issues, the Comprehensive Complication Index (CCI) was introduced, integrating all complications into a single severity-weighted score [6]. In a previous proof-of-concept study, we showed that the combination of the EAU reporting criteria and the CCI improved estimates of 30-d morbidity after RC [1]. This framework has since been applied to other urological procedures [[7], [8], [9]], but all prior studies were retrospective, leaving key questions unresolved. One critical gap concerns how perioperative morbidity affects health-related quality of life (HRQOL) after RC [10]. Although a relationship seems intuitive, few studies have directly explored it, and none used standardized tools for both complication grading and patient-reported outcomes. A recent UK study involving 20 000 patients undergoing major abdominal surgery found a significant association between postoperative complications and lower HRQOL for up to 12 mo [11], with greater severity linked to worse outcomes. While RC is known to temporarily impair HRQOL—especially physical, mental, and social domains [12]—the impact of complication severity on long-term HRQOL remains unclear [13]. We therefore initiated a prospective study with three objectives: (1) to systematically assess 90-d morbidity after RC, (2) to evaluate peri- and postoperative HRQOL, and (3) to examine the relationship between cumulative complication burden and HRQOL. We here present the first results from the Comprehensive Outcome Measures and Perioperative Morbidity After CystecTomy (COMPACT) registry.

## 2 Patients and methods

### 2.1 Study population

COMPACT is a prospective investigation (German Clinical Trials DRKS00024929). It comprises clinical, pathological, morbidity, HRQOL, and oncological outcome data from our tertiary referral center. The study includes patients who underwent open RC with bilateral pelvic lymph node dissection for histologically confirmed bladder cancer. Starting from May 1, 2020, all eligible patients were consecutively approached for study participation. Indication and pre-, intra-, and postoperative workflow have been described in detail previously (Supplementary material) [1]. All surgeries were performed by one of three senior surgeons, each with several years of experience in RC. Although the study is ongoing, for the analysis at hand, patients were included up until May 31, 2022, to ensure an adequate follow-up period. A total of four individuals were excluded from the study due to their presentation with conditions other than bladder cancer, while ten individuals refused to participate in the study. Additionally, nine patients completed only the preoperative questionnaire and were subsequently lost to follow-up (Supplementary Fig. 1). The study was approved by the local ethics committee of the medical council of Hamburg (no. PV5634).

### 2.2 Definition, extraction, and grading of complications

Definition of the complication catalog followed our proof-of-concept study [1]. Most importantly, we formulated explicit definitions for each potential adverse event by utilizing the Common Terminology Criteria for Adverse Events version 5.0. These definitions were established after a thorough review of the literature, incorporating landmark studies pertaining to morbidity assessment following RC. Complications were ascertained from the following sources: digitalized charts (Soarian Clinicals), scheduled phone calls, and questionnaires administered to the patients at 30- and 90-d intervals postoperatively. The objective was to evaluate any complications occurring within 90 d after discharge, which might not have been documented in the hospital charts. The process for collecting and grading complications involved several steps. First, any complication occurring within 90 d after RC was documented for each patient, and then each complication was categorized according to the validated and adapted CDC [3,4]. “Minor” complications were defined as CDC grades ≤IIIa, while “major” complications were classified as CDC grades ≥IIIb [1]. Second, the 90-d CCI was calculated for each patient using the online calculator available at https://www.cci-calculator.com. The CCI, which ranges from 0 (indicating an uneventful course) to 100 (representing death), synthesizes cumulative perioperative morbidity [6].

### 2.3 Patient-reported outcome measures to assess HRQOL

HRQOL was evaluated using a validated patient-reported outcome measure (PROM): the German version of the Functional Assessment of Cancer Therapy—Bladder—Cystectomy (FACT-BL-CYS) questionnaire [14,15]. The FACT-BL-CYS questionnaire utilizes a 5-point Likert scale, ranging from 0 to 4, to assess various facets of HRQOL. These responses contribute to subdomains related to physical well-being, social/family well-being, emotional well-being, and functional well-being. In turn, these subdomains comprise the Functional Assessment of Cancer Therapy—General (FACT-G) total score, which ranges from 0 to 108, representing an overall summary of HRQOL. Moreover, there is an ancillary bladder/cystectomy (BL-CYS) subscale, ranging from 0 to 60, which captures specific issues related to RC. This subscale provides a more nuanced understanding of the disease-specific impacts on HRQOL. Lastly, the FACT-BL-CYS questionnaire computes an overall score by combining all subdomains, represented as the FACT-BL-CYS total score, which ranges from 0 to 168. A higher score on this scale signifies better HRQOL.

HRQOL was evaluated at various intervals: preoperatively (baseline) and at 3, 6, and 12 mo postoperatively. The primary endpoint, measured at the 6-mo postoperative mark, was the HRQOL quantified by the FACT-BL-CYS total score. The 6-mo follow-up was selected as the primary time point for HRQOL assessment, based on prior findings indicating that most patients achieve substantial recovery within this timeframe following RC [12], and that global HRQOL exceeds baseline levels by 6 mo in key studies [16]. The secondary endpoints included the FACT-G total score and the BL-CYS subscale.

### 2.4 Clinical, surgical, and pathological characteristics

We comprehensively assessed clinical features including age, sex, body mass index (BMI), age-adjusted Charlson Comorbidity Index (ACCI) [17,18], smoking status, preoperative hydronephrosis, preoperative hemoglobin levels, and estimated glomerular filtration rate using the Chronic Kidney Disease Epidemiology Collaboration equation [19]. Furthermore, this study considered chronic kidney disease stage based on National Kidney Foundation guidelines, a prior history of pelvic or abdominal surgeries and radiotherapy, and previous systemic chemotherapy for non–bladder cancer malignancies. Surgical characteristics were also documented, such as operative time, type of urinary diversion performed, and administration of neoadjuvant chemotherapy for bladder cancer. Pathological features included the most recent tumor, node, and metastasis classification system, specifically examining tumor and nodal stages. Lastly, we evaluated whether adjuvant systemic therapy was administered and identified the type of treatment provided, distinguishing between chemotherapy and immunotherapy. Table 1 provides a detailed overview of these attributes.

**Table 1:** Clinical, surgical, and pathological characteristics of 82 patients who underwent open radical cystectomy for bladder cancer between May 2020 and May 2022

	Overall	ACCI ≤2	ACCI 3–5	ACCI ≥6
Patients, *n* (%)	82 (100)	26 (32)	46 (56)	10 (12)
Age (yr), median (IQR)	68 (62–73)	59 (57–63)	72 (66–76)	73 (71–77)
Sex: male, *n* (%)	60 (73)	14 (54)	39 (85)	7 (70)
Body mass index, median (IQR)	26 (24–29)	26 (23–29)	27 (24–28)	26 (24–28)
Smoking status, *n* (%)				
Never	15 (18)	5 (19)	10 (22)	0 (0)
Former	47 (57)	12 (46)	32 (70)	3 (30)
Current	20 (24)	9 (35)	4 (8.7)	7 (70)
Hydronephrosis, *n* (%)	11 (13)	3 (12)	6 (13)	2 (20)
Hemoglobin (g/dl), median (IQR)	13 (12–14)	14 (12–15)	13 (12–14)	13 (11–14)
eGFR (ml/min/1.73 m^2^), median (IQR)	71 (62–87)	80 (67–92)	69 (61–85)	65 (52–84)
CKD stage according to the NKF, *n* (%)				
Stage 1–2	65 (79)	23 (88)	36 (78)	6 (60)
Stage 3–5	17 (21)	3 (12)	10 (22)	4 (40)
Prior pelvic or abdominal radiotherapy, *n* (%)	8 (9.8)	0 (0)	6 (13)	2 (20)
Prior systemic chemotherapy a,*n* (%)	2 (2.4)	1 (3.9)	1 (2.2)	0 (0)
Neoadjuvant chemotherapy, *n* (%)	8 (9.8)	3 (12)	5 (11)	0 (0)
Operative time (min), median (IQR)	305 (237–345)	336 (266–371)	305 (230–331)	267 (211–310)
Continent urinary diversion, *n* (%)	17 (21)	12 (46)	5 (11)	0 (0)
Urinary diversion subtype, *n* (%)				
Neobladder	10 (12)	7 (27)	3 (6.5)	0 (0)
Ileal conduit	46 (56)	12 (46)	28 (61)	6 (60)
Mainz pouch I	7 (8.5)	5 (19)	2 (4.4)	0 (0)
Colon conduit	1 (1.2)	0 (0)	1 (2.2)	0 (0)
Cutaneous ureterostomy	17 (21)	2 (7.7)	11 (24)	4 (40)
No diversion/dialysis	1 (1.2)	0 (0)	1 (2.2)	0 (0)
Length of stay (d), median (IQR)	17 (15–21)	18 (15–22)	17 (15–20)	17 (12–20)
pT stage, *n* (%)				
pT0	11 (13)	5 (19)	6 (13)	0 (0)
pTa	5 (6.1)	0 (0)	5 (11)	0 (0)
pTis	9 (11)	3 (12)	4 (8.7)	2 (20)
pT1	9 (11)	3 (12)	5 (11)	1 (10)
pT2	19 (23)	8 (31)	8 (17)	3 (30)
pT3	21 (26)	6 (23)	11 (24)	4 (40)
pT4	8 (9.8)	1 (3.9)	7 (15)	0 (0)
pN stage, *n* (%)				
pN0	63 (77)	19 (73)	35 (76)	9 (90)
pN+	19 (23)	7(27)	11 (24)	1 (10)
Organ confinement, *n* (%)				
≤pT2 and pN0	47 (57)	15 (58)	26 (57)	6 (60)
pT3 and/or pN+	35 (43)	11 (42)	20 (43)	4 (40)
Adjuvant systemic treatment, *n* (%)	24 (29)	8 (31)	13 (28)	3 (30)
Type of adjuvant systemic treatment (*n* = 24), *n* (%)				
Chemotherapy	17 (71)	7 (88)	9 (69)	1 (33)
Immunotherapy	7 (29)	1 (13)	4 (31)	2 (67)

ACCI = age-adjusted Charlson Comorbidity Index; CKD = chronic kidney disease; eGFR = estimated glomerular filtration rate; IQR = interquartile range; NKF = National Kidney Foundation.

Percentages may not add up to 100%, as these are rounded.

a Refers to chemotherapy administered for malignancies other than bladder cancer.

### 2.5 Statistical analyses

First, we conducted descriptive analyses to characterize our study population undergoing RC by examining clinical, surgical, and pathological features. To delineate the impact of comorbidity burden, we stratified our cohort based on ACCI cutoffs validated previously for RC into three groups: ≤2, 3–5, and ≥6 [18]. Second, in order to adhere to the quality criteria laid out by the EAU for standardized reporting, we defined and reported three key morbidity estimates: overall complications, most severe complications (CDC grades IIIb–IV), and mortality (CDC grade V) [1,2]. Third, we calculated the FACT-BL-CYS total score and its subscales, the FACT-G total score, and the BL-CYS subscale at each time point using the manual scoring template. We used the Wilcoxon signed-rank test to compare FACT-BL-CYS total scores between adjacent time points to assess changes over time. In addition, we conducted linear regression analyses to identify factors associated with our main endpoint HRQOL at 6 mo postoperatively. Missing data were handled by a complete case analysis, that is, without imputation, consistent with prior studies in this setting. Linear regression analyses evaluated our main endpoint using (1) 90-d CDC grade ≥IIIb versus ≤IIIa and (2) 90-d CCI as predictors. Models were adjusted for ACCI, sex, BMI, continent versus incontinent urinary diversion, organ confinement (≤pT2N0 vs ≥pT3 or N+), and receipt of adjuvant treatment. To evaluate the potential attrition bias due to missing data, baseline characteristics—including age, sex, ACCI strata, and diversion type—were compared between patients with available 6-mo HRQOL data and those with missing data.

All statistical analyses were performed using Stata (release 17; StataCorp LP, College Station, TX, USA). The reported *p* values were two sided, and *p* values <0.05 were considered statistically significant.

## 3 Results

### 3.1 Descriptive analyses of clinical, surgical, and pathological characteristics

Of 105 patients, 82 (78%) had PROMs available at least at three of the four investigated time points, including baseline and 3, 6, and 12 mo postoperatively. The median age was 68 yr (interquartile range [IQR] 62–73). Most patients were male (60, 73%), and the median baseline ACCI was 3 (IQR 2–5). The median operative time was 305 min (IQR 237–345). A total of 46 patients (56%) underwent RC with an ileal conduit, followed by 17 (21%) with a cutaneous ureterostomy and ten (12%) with a neobladder. Disease stage distribution included 47 patients (57%) with localized disease (pT2N0) and 35 (43%) with locally advanced disease (pT3 and/or pN+). Across ACCI strata, a larger proportion of men than women were observed in the higher comorbidity groups, and the frequency of continent diversions decreased with increasing ACCI scores. Table 1 summarizes all patient characteristics and their categorization.

### 3.2 Assessment of perioperative 90-d complications

**Fig. 1:**
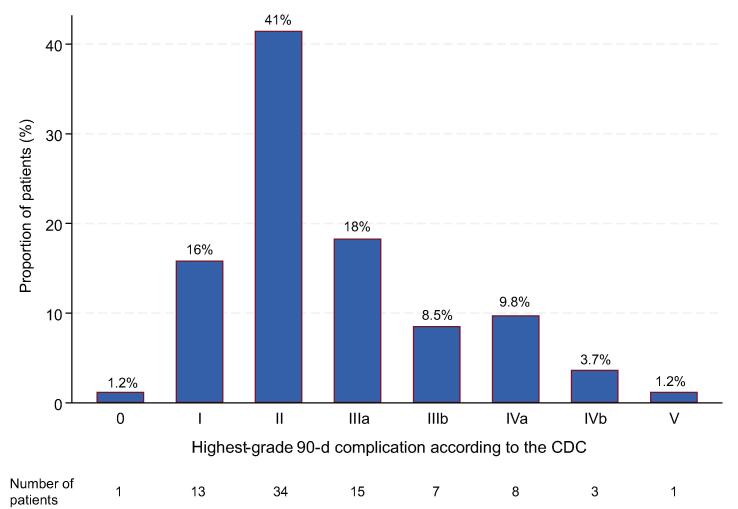
Column charts depicting the distribution of the highest-grade 90-d complications per patient after open radical cystectomy with pelvic lymph node dissection and urinary diversion stratified by the Clavien-Dindo classification (CDC).

A detailed summary of the numbers and proportions of all recorded complication types, categories, and CDC grading is provided in Table 2. A total of 572 complications occurred in 81 of 82 patients (99%; 95% confidence interval [CI] = 93–100%) within the first 90 d postoperatively, corresponding to a median number of 7 (IQR 5–9) complications per patient. Of these patients, 47 (57%) experienced CDC grade I–II complications, 22 (27%) had CDC grade III complications, and 11 (13%) had CDC grade IV complications (Fig. 1). In total, 18 patients (22%; 95% CI = 14–32%) developed most severe complications (CDC grades IIIb–IV). The 90-d mortality rate (CDC grade V) was 1.2% (95% CI = 0.03–6.6%).

**Table 2:** Frequencies, proportions, therapeutic management, and grading of perioperative 90-d complications in 82 patients who underwent open radical cystectomy between May 2020 and May 2022

		CDC grading	Management	Number of complications	Proportion, % (*N* = 82)
*Gastrointestinal*					
101 complications (18%) a					
	Ileus (paralytic)	I	Conservative; cessation of oral intake and i.v. fluid support	2	2.4
		II	Conservative; antibiotic treatment, analgesic treatment, bowel stimulation	1	1.2
		IIIa	Replacement of nasogastric tube	4	4.9
		IIIb	Laparotomy	2	2.4
	Small bowel obstruction (mechanical)	IIIb	Laparotomy	2	2.4
	Constipation	I	Conservative; laxatives, i.v. fluid support	34	41
		IIIa	Nasogastric tube/intestinal tube	1	1.2
	*Clostridium difficile* colitis	II	Antibiotic treatment	3	3.7
	Gastrointestinal bleeding	I	Conservative; clinical observation or diagnostic evaluation only	0	0
		II	Blood transfusion	0	0
	Emesis	I	Conservative; antiemetics and i.v. fluid support	31	38
		IIIa	Nasogastric tube	5	6.1
	Anastomotic bowel leak	I	Conservative; antiemetics and i.v. fluid support	2	2.4
		IIIa	Nasogastric tube	1	1.2
		IIIb	Laparotomy	1	1.2
	Diarrhea (*C. difficile* associated)	I	Conservative; antidiarrheals, i.v. fluid support, electrolytes	12	15
*Infectious*					
87 complications (15%) a					
	Fever of unknown origin	I	Conservative; i.v. fluid support	1	1.2
		II	Conservative; antipyretics, antibiotic treatment	3	3.7
	Bacteriuria (>10^5^ cfu/ml; asymptomatic)	I	Conservative; no antibiotic treatment	25	30
		II	Antibiotic treatment	1	1.2
	Urinary tract infection (>10^5^ cfu/ml; symptomatic)	II	Antibiotic treatment	43	52
	Abscess	IIIa	Incision and drainage	1	1.2
	Sepsis (SIRS in response to infectious process)	II	Antibiotic treatment, supportive care	1	1.2
		IVa	Septic single organ dysfunction, ICU	1	1.2
		IVb	Septic multiorgan dysfunction, ICU	2	2.4
	Urosepsis	II	Antibiotic treatment	1	1.2
		IIIa	Nephrostomy	2	2.4
		IVa	Septic single organ dysfunction, ICU	1	1.2
	Pyelonephritis	II	Antibiotic treatment	2	2.4
		IIIa	Nephrostomy	1	1.2
	Diverticulitis	II	Antibiotic treatment	0	0
	Gastroenteritis	II	Conservative; antiemetics, antibiotic treatment	0	0
	Cholecystitis	IIIb	Cholecystectomy	2	2.4
*Wound*					
21 complications (3.7%) a					
	Wound seroma	I	Conservative; clinical observation or diagnostic evaluation only	1	1.2
	Wound infection (SSI)	I	Conservative; clinical observation or diagnostic evaluation only	2	2.4
		II	Antibiotic treatment	3	3.7
	Wound dehiscence (fascia intact)	I	Conservative; clinical observation or diagnostic evaluation only, reinforced adhesive skin closure	10	12
		IIIb	Secondary surgical closure	1	1.2
	Fascial dehiscence/evisceration	IIIb	Secondary surgical closure	4	4.9
*Genitourinary*					
54 complications (9.4%) a					
	Acute kidney injury	I	Conservative; i.v. fluid support, diuretics	3	3.7
		II	Antibiotic treatment	1	1.2
		IVa	Dialysis	4	4.9
	Hydronephrosis (new onset)	I	Conservative; clinical observation or diagnostic evaluation only	30	37
	Ureteral obstruction	I	Conservative; clinical observation or diagnostic evaluation only	2	2.4
		IIIa	Nephrostomy	2	2.4
	Urinary leak/urinoma	I	Conservative; clinical observation or diagnostic evaluation only, deferred Foley catheter extraction	2	2.4
		IIIa	Drainage	1	1.2
		IIIb	Ureter reimplantation/retrograde ureteral stenting	2	2.4
	Urinary retention	I	Conservative; clinical observation or diagnostic evaluation only, replacement of Foley catheter (neobladder/pouch)	1	1.2
	Parastomal hernia	I	Conservative; clinical observation or diagnostic evaluation only	0	0
		IIIb	Laparotomy and surgical revision	0	0
	Urostomy ischemia	I	Conservative; clinical observation or diagnostic evaluation only	0	0
		IIIb	Laparotomy and surgical revision	0	0
	Hematuria	I	Conservative; clinical observation or diagnostic evaluation only	6	7.3
*Cardiac*					
31 complications (5.4%) a					
	Arrhythmia	II	Conservative; medical cardioversion	1	1.2
		IIIb	Pacemaker, cardioversion	1	1.2
	Myocardial infarction	IV	Coronary angiography, stent implantation, ICU	0	0
	Hypertension (new onset)	I	Conservative; clinical observation or diagnostic evaluation only	3	3.7
		II	Antihypertensives	2	2.4
	(Acute) congestive heart failure	IVa	Coronary angiography, ICU	0	0
	Angina (pectoris)	I	Conservative, clinical observation or diagnostic evaluation only	0	0
	Hypotension	I	Conservative; i.v. fluid support	21	26
		II	Medical treatment	3	3.7
*Pulmonary*					
49 complications (8.6%) a					
	Atelectasis	II	Continuous positive airway pressure, physiotherapy	1	1.2
	Pneumonia	II	Antibiotic therapy	5	6.1
		IVa	Single organ dysfunction, ICU	1	1.2
	Respiratory distress/dyspnea	I	Oxygen, physiotherapy	38	46
		IVb	Multiple organ dysfunction, ICU	1	1.2
	Pneumothorax	I	Conservative; clinical observation or diagnostic evaluation only	0	0
		IIIa	Chest tube	0	0
	Pleural effusion	I	Conservative; clinical observation or diagnostic evaluation only	2	2.4
		IIIa	Chest tube	1	1.2
*Bleeding*					
19 complications (3.3%) a					
	Anemia requiring transfusion	I	Conservative; clinical observation, patient refused transfusion	1	1.2
		II	Blood transfusion	12	15
	Postoperative bleeding other than gastrointestinal	I	Conservative; clinical observation or diagnostic evaluation only	0	0
		II	Blood transfusion/fibrinogen	0	0
		IIIa	Suture ligation	0	0
		IIIb	Laparotomy and surgical revision	0	0
	Wound hematoma	I	Conservative; clinical observation or diagnostic evaluation only	5	6.1
		IIIa	Drainage	1	1.2
*Thromboembolic*					
10 complications (1.7%) a					
	Deep vein thrombosis	II	Anticoagulation	4	4.9
		IIIa	Angioplasty	1	1.2
	Pulmonary embolism	II	Anticoagulation	3	3.7
		IVa	ICU, anticoagulation	1	1.2
	Superficial phlebitis	I	Conservative; clinical observation	1	1.2
*Neurological*					
39 complications (6.8%) a					
	Peripheral neuropathy	I	Conservative; clinical observation or diagnostic evaluation only	8	9.8
	CVA/TIA	II	Antiplatelets, anticoagulation	1	1.2
	Delirium/agitation	I	Treatment of underlying main complication	5	6.1
		II	Antipsychotics	2	2.4
	Vertigo	I	Conservative; i.v. fluid support	18	22
	Loss of consciousness/syncope	I	Conservative; clinical observation or diagnostic evaluation only	5	6.1
	Seizure	II	Medical therapy	0	0
*Intraoperative*^b^					
0 complications (0%) a					
	Vascular injury	–	–	0	0
	Bowel injury	–	–	0	0
	Retained foreign body	–	–	0	0
*Miscellaneous*					
161 complications (28%) a					
	Psychological illness	I	Conservative; psycho-oncological support	17	21
		II	Antidepressant drugs	2	2.4
	Dermatitis	I	Ointment	1	1.2
	Acidosis	I	Conservative; medical therapy, electrolytes	0	0
	Thrombocytopenia	I	Conservative	1	1.2
	Decubitus ulcer	I	Conservative	8	9.8
	Lymphocele	I	Conservative; clinical observation or diagnostic evaluation only	1	1.2
		II	Conservative; antibiotic treatment	1	1.2
		IIIa	Drainage	2	2.4
		IIIb	Drainage under general anesthesia	2	2.4
	Dehydration	I	Conservative; i.v. fluid support	45	55
	Edema	I	Conservative; medical therapy	38	46
	Hypokalemia	I	Conservative; medical therapy	34	41
	Other rare complications	I	Conservative	6	7.3
		II	Conservative; medical therapy	3	3.7
*Mortality*					
1 complication (0.17%)					
	Tumor progression, DOD	V	Palliative care	1	1.2

CDC = Clavien-Dindo classification; cfu = colony-forming units; CVA = cerebrovascular accident; DOD = dead of disease; ICU = intensive care unit; i.v. = intravenous; SIRS = systemic inflammatory response syndrome; SSI = surgical site infection; TIA = transient ischemic attack.

Percentages may not add up to 100%, as these are rounded.

a The percentage refers to the proportion of all 572 complications.

b The CDC does not apply to intraoperative complications. Thus, no grading system was used.

The median 90-d CCI was 35 (IQR 26–45). The most common complications were gastrointestinal (18%), followed by infectious complications (15%; see Table 2). Genitourinary and pulmonary complications occurred in 9.4% and 8.6%, respectively, while neurological complications were reported in 6.8%. Cardiac (5.4%), wound (3.7%), bleeding (3.3%), and thromboembolic (1.7%) complications were rare. Among miscellaneous complications, dehydration, edema, and hypokalemia were most prevalent, collectively accounting for 82% of this category. Supplementary Figure 2 displays the proportions of all 90-d complication subgroups relative to the total number of recorded complications, as well as the proportion of Clavien-Dindo grade ≥IIIb events within each subgroup.

### 3.3 HRQOL over the perioperative course

**Fig. 2:**
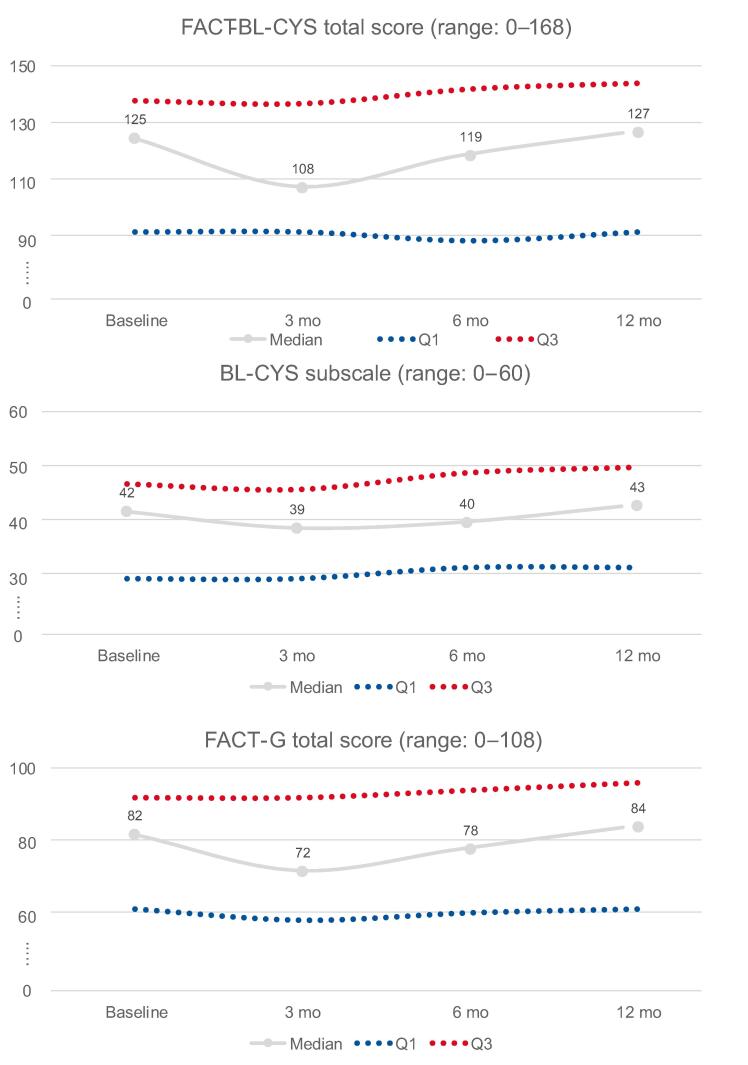
Median health-related quality of life (HRQOL) scores at baseline and 3, 6, and 12 mo in patients who underwent open radical cystectomy with pelvic lymph node dissection and urinary diversion. FACT-BL-CYS = Functional Assessment of Cancer Therapy—Bladder—Cystectomy; FACT-G = Functional Assessment of Cancer Therapy—General.

Median FACT-BL-CYS total scores were lowest at 3 mo postoperatively. However, paired comparisons using the Wilcoxon signed-rank test did not show a significant difference between baseline and 3 mo (*p* = 0.5), indicating no consistent directional change across individuals. A significant improvement was observed between 3 and 6 mo (*p* = 0.009), while scores remained stable between 6 and 12 mo (*p* = 0.6). The median FACT-BL-CYS total score decreased from 125 (IQR 93–138) at baseline to 108 (93–137) at 3 mo, improved to 119 (90–142) at 6 mo, and returned to 127 (93–144) at 12 mo. A similar trend was observed for the FACT-G total score (82 → 72 → 78 → 84) and the BL-CYS subscale (42 → 39 → 40 → 43; Fig. 2). Questionnaire response rates remained high over time: 96% at baseline, 90% at 3 mo, 87% at 6 mo, and 78% at 12 mo. Baseline characteristics were similar between patients with and without 6-mo HRQOL data, with no significant differences in age (*p* = 0.4), sex (*p* > 0.9), ACCI strata (*p* = 0.4), or diversion type (*p* > 0.9).

### 3.4 Evaluation of the HRQOL and association with CDC and CCI

**Fig. 3:**
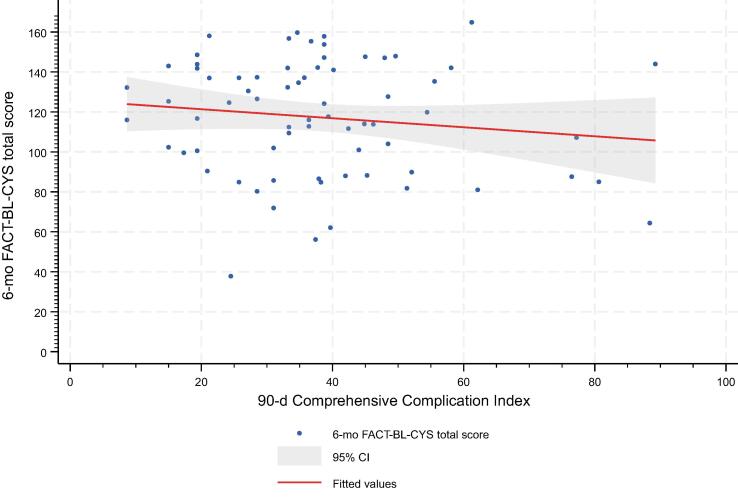
Scatter plot of 90-d CCI versus 6-mo FACT-BL-CYS total score. Each point represents an individual patient. The red line indicates the fitted linear regression, with the shaded area representing the 95% confidence interval. The nearly flat slope suggests little or no linear association between perioperative complications and HRQOL at 6 mo. CCI = Comprehensive Complication Index; CI = confidence interval; FACT-BL-CYS = Functional Assessment of Cancer Therapy—Bladder—Cystectomy; HRQOL = health-related quality of life.

Two multivariable linear regression analyses were modeled investigating 90-d CDC ≥IIIb and the 90-d CCI as predictors for the 6-mo FACT-BL-CYS total score (Table 3). After adjusting for baseline characteristics, tumor parameters, and adjuvant treatment, we did not observe a statistically significant association of 90-d CDC grade ≥IIIb (vs CDC grade ≤IIIa; *p* = 0.7) or the 90-d CCI with 6-mo HRQOL (*p* = 0.2). Indeed, comorbidity was the driving factor of our endpoint showing the ACCI as a significant predictor in both models; each 1-point increase in the ACCI corresponded to a 4.6–4.7-point decrease in the FACT-BL-CYS total score (both *p* = 0.02). The relationship between the 90-d CCI and 6-mo FACT-BL-CYS total score was further visualized using a scatter plot (Fig. 3).

**Table 3:** Multivariable linear regression models exploring potential associations of clinical characteristics and short-term morbidity after open radical cystectomy with 6-mo health-related quality of life, as measured by the FACT-BL-CYS total score

	Model 1: 90-d CCI		Model 2: 90-d CDC ≥IIIb	
	Coefficient (95% confidence intervals)	*p* value	Coefficient (95% confidence intervals)	*p* value
*Multivariable linear regression: 6-mo FACT-BL-CYS total score*				
90-d CCI; continuous per 10 points	–2.9 (–7.4; 1.6)	0.2	–	–
90-d CDC ≥IIIb	–	–	–3.3 (–22; 15)	0.7
ACCI; continuous	–4.6 (–8.4; –0.82)	0.02	–4.7 (–8.6–; –0.91)	0.02
Female sex	–4.2 (–22; 13)	0.6	–3.5 (–21; 14)	0.7
BMI; continuous per 5 kg/m^2^	2.7 (–5.7; 11)	0.5	1.4 (–6.9; 9.6)	0.7
Continent urinary diversion	–9.7 (–29; 9.8)	0.3	–13 (–32; 5.8)	0.2
Organ confinement: pT3 and/or pN+	–9.5 (–28; 8.5)	0.3	–9.8 (–28; 8.5)	0.3
Adjuvant systemic treatment	1.9 (–18; 22)	0.8	2.7 (–17; 23)	0.8

ACCI = age-adjusted Charlson comorbidity index; BMI = body mass index; CCI = Comprehensive Complication Index; CDC = Clavien-Dindo classification; FACT-BL-CYS = Functional Assessment of Cancer Therapy—Bladder—Cystectomy.

## 4 Discussion

Standardized and meticulous complication reporting after major urological surgery is essential for comparing outcomes across institutions, surgical approaches, and patient-reported HRQOL. In a previous retrospective study, we demonstrated that adherence to EAU reporting guidelines and the use of the CCI improved the estimation of perioperative morbidity after RC [1]. However, that analysis lacked prospective data and HRQOL outcomes. In the current study, we addressed these gaps with a prospective design and present the first results from the COMPACT registry. Consistent with our prior findings, nearly all patients (99%) experienced at least one complication within 90 d after RC, confirming the high morbidity associated with this procedure. However, most adverse events were minor. Importantly, we did not find statistically significant associations between major complications or cumulative morbidity, as measured by the CDC or CCI, and 6-mo HRQOL, although the wide CIs indicate that some uncertainty persists. Instead, age-adjusted comorbidity emerged as the only independent predictor of HRQOL.

The slightly higher morbidity burden observed in this prospective 90-d assessment compared with our earlier 30-d analysis is likely explained by three main factors. First, the prospective nature of our study enabled rigorous and systematic complication reporting. Patients were monitored daily during hospitalization, and complications were documented using a predefined, comprehensive catalog [1]. A structured approach such as this increases the detection of complications, particularly minor complications. We argue that true 90-d morbidity remains under-reported in the literature due to postdischarge follow-up challenges, particularly in decentralized health systems such as those of Germany. Our study minimized such data loss through scheduled telephone follow-ups and structured morbidity questionnaires, ensuring a high level of completeness and accuracy in complication capture. Second, the extended 90-d observation period naturally results in a higher cumulative complication rate than shorter follow-up durations. This reinforces the importance of longer-term tracking to more accurately reflect perioperative morbidity. Third, underestimation of low-grade complications is a common issue in previous studies, particularly in urology [20]. Our use of the CCI allowed for nuanced grading of cumulative morbidity, acknowledging that minor complications—although unlikely to impact recovery individually—still contribute to the overall burden. This approach aligns with other prospectively collected series that report major complication rates of approximately 20% after RC [21]. We are therefore confident that the complication spectrum and intensity reported in the COMPACT registry reflect accurately the real-world morbidity associated with open RC.

Bladder cancer diagnosis and RC are known to impair HRQOL across physical, emotional, and social dimensions [22]. However, prior studies show that HRQOL often recovers by 3–12 mo postoperatively [16,[23], [24], [25], [26], [27]]. Our data confirm this trajectory: 6-mo HRQOL, measured via the FACT-BL-CYS total score, did not differ from baseline values. We found no statistically significant associations between HRQOL and either major complications or cumulative morbidity. The wide CIs, however, indicate considerable uncertainty, and thus a clinically relevant effect of perioperative morbidity on HRQOL cannot be excluded. Overall, our results align with prior studies showing that complications may affect specific HRQOL subdomains but have limited influence on overall HRQOL [28]. Several explanations support this finding. First, patients undergoing RC often exhibit remarkable resilience, aided by structured perioperative care and rehabilitation, which facilitates recovery even after complications. Second, other factors—particularly pre-existing comorbidities and individual psychological adaptation—may exert a greater influence on HRQOL outcomes than perioperative complications. This is reflected in our multivariable analysis, where comorbidity was the only independent predictor of 6-mo HRQOL. Although no minimally important difference has yet been established for the FACT-BL-CYS, estimates can be derived from related instruments. For instance, a study on the FACT-Colorectal reported that a 5–8 point difference is clinically meaningful [29], and the Functional Assessment of Chronic Illness Therapy guidelines suggest that a change of approximately 0.15 points per item can be regarded as the minimally important difference [30], which for the 42-item FACT-BL-CYS corresponds to about 6 points. This indicates that the observed decrease of 4–5 points per additional ACCI point is close to, and may well represent, a clinically meaningful effect. Collectively, these insights underscore the multifactorial nature of postoperative recovery and highlight the importance of individualized patient care. While short-term complications remain clinically important, these do not appear to determine longer-term quality of life. These findings can reassure patients and guide clinicians in preoperative counseling and expectation management.

The strengths of our study include its prospective design, accurate 90-d complication assessment based on a validated methodology, and use of a robust, disease-specific PROM for HRQOL. However, several limitations merit discussion. First, while intraoperative complications may influence postoperative recovery, we did not apply a standardized classification system (eg, ICARUS) [31] and may have under-reported such events, although no major intraoperative adverse events were observed. Nonetheless, the comprehensive 90-d follow-up likely captured clinically relevant sequelae. Second, our analysis was limited to patients undergoing open RC. While robotic techniques have gained increasing popularity, especially in specialized centers, open RC remains the predominant approach in many health care systems, including those in Germany, where it still accounts for the majority of cases. Moreover, available randomized evidence—most notably the Memorial Sloan Kettering Cancer Center trial, the only trial powered to detect differences in 90-d complications [32]—has demonstrated comparable 90-d morbidity between open and robotic approaches. Given the similar perioperative morbidity profiles when applying standardized complication reporting [33], comparable findings regarding the association between complications and HRQOL may reasonably be expected in robotic series as well. Nevertheless, future studies should apply the same standardized methods to robotic RC to validate and extend these findings. Third, this was a single-center study. While it ensured high data quality, the findings require validation in multicenter settings to enhance generalizability. Fourth, important factors such as patient resilience and psychosocial characteristics—which may logically influence HRQOL after major surgery—were not captured in our analysis. We acknowledge this as a limitation that warrants further investigation. Fifth, our focus on 6-mo HRQOL may not fully capture the long-term trajectory of HRQOL, particularly in patients with continent urinary diversions. These patients often require a longer adaptation period for functional and psychosocial adjustment, and HRQOL domains may continue to evolve beyond the early postoperative phase. While the 6-mo time point was selected based on prior studies demonstrating substantial recovery and normalization of global HRQOL by this time [16], we acknowledge that longer-term data are essential to characterize postoperative HRQOL more comprehensively. As additional follow-up data become available within the registry, future analyses will address extended outcomes, including time-dependent and diversion-specific trends. Sixth, missing PROM data may have influenced the results. Of 105 eligible patients, 82 contributed PROMs overall and 71 had evaluable 6-mo FACT-BL-CYS data, forming the denominator for the regression analyses. A comparison of baseline characteristics revealed no significant differences between patients with and without the 6-mo data, suggesting a limited risk of a systematic attrition bias. Nevertheless, we acknowledge that loss to follow-up could still have influenced the results, particularly if patients with poorer health status were less likely to complete PROMs. Lastly, we acknowledge the absence of formal power analyses, which is common in multivariable models within observational studies—even those with prospectively collected data—since such models primarily serve to adjust for confounding rather than to test multiple hypotheses. We agree that a substantial increase in the sample size, thereby including more patients with CDC grade ≥IIIb complications, could potentially impact the results. The CIs are provided to convey the precision of our estimates transparently, which indicate some persisting uncertainty in effect sizes.

In summary, our findings highlight that although RC is associated with high perioperative morbidity, this does not necessarily translate into reduced HRQOL at 6 mo. These results underscore the importance of preoperative comorbidity and holistic perioperative care in shaping patient-centered outcomes.

## 5 Conclusions

This prospective analysis from the COMPACT registry underscores the importance of rigorous, standardized assessment of perioperative morbidity following RC. While complications remain nearly universal, most are of low clinical severity and do not appear to diminish HRQOL at 6 mo postoperatively. Instead, patient-specific factors such as comorbidity seem to play a more decisive role in long-term well-being. These findings suggest that surgical morbidity alone may not fully explain variations in postoperative recovery, emphasizing the need for a holistic approach to perioperative care that includes targeted pre- and postoperative rehabilitation, psychosocial support, and individualized patient counseling. Future research should further explore these dynamics across surgical modalities and care settings to improve outcomes beyond traditional morbidity metrics.

  ***Author contributions*:** Malte W. Vetterlein had full access to all the data in the study and takes responsibility for the integrity of the data and the accuracy of the data analysis.

  *Study concept and design*: Koelker, Klemm, von Deimling, Vetterlein.

*Acquisition of data*: Trepte, Kukuk, Wirtz.

*Analysis and interpretation of data*: Koelker, Vetterlein.

*Drafting of the manuscript*: Koelker, Trepte, Vetterlein.

*Critical revision of the manuscript for important intellectual content*: Klemm, von Deimling, Kukuk, Wirtz, Pachollek, Luebbersmeyer, Ludwig, Dahlem, Fisch.

*Statistical analysis*: Koelker, Vetterlein.

*Obtaining funding*: None.

*Administrative, technical, or material support*: None.

*Supervision*: None.

*Other*: None.

  ***Financial disclosures:*** Malte W. Vetterlein certifies that all conflicts of interest, including specific financial interests and relationships and affiliations relevant to the subject matter or materials discussed in the manuscript (eg, employment/affiliation, grants or funding, consultancies, honoraria, stock ownership or options, expert testimony, royalties, or patents filed, received, or pending), are the following: None.

  ***Funding/Support and role of the sponsor*:** None.
