# Supplementary material for: The Interplay of Cumulative Perioperative Morbidity and Health-related Quality of Life After Radical Cystectomy—Prospective Evidence from the COMPACT Registry
Source: Eur Urol Open Sci. 2025 Oct 11;82:13–24. doi: 10.1016/j.euros.2025.09.013 (PMC12547919; doi:10.1016/j.euros.2025.09.013)
Supplement: Supplementary Data 1 [file mmc1.docx]

**Supplementary Figure 1 –** Diagram illustrating the selection process for patients included in the final analyses.

* Three patients did not respond to the preoperative questionnaire, but they did complete all other questionnaires.

**Supplementary Figure 2** – Radar plot illustrating the relative proportions of all 90-day postoperative complication subgroups based on the total number of recorded complications. The plot also shows the proportion of complications classified as Clavien-Dindo grade ≥ IIIb within each subgroup. Note: Proportions of intraoperative complications are not shown, as the Clavien-Dindo classification does not apply to events occurring during surgery.
