# Supplementary material for: The Interplay of Cumulative Perioperative Morbidity and Health-related Quality of Life After Radical Cystectomy—Prospective Evidence from the COMPACT Registry
Source: Eur Urol Open Sci. 2025 Oct 11;82:13–24. doi: 10.1016/j.euros.2025.09.013 (PMC12547919; doi:10.1016/j.euros.2025.09.013)

Supplementary Methods

Indication, Preoperative, Intraoperative, and Postoperative Workflow in Patients Undergoing Open Radical Cystectomy at the Department of Urology, University Medical Center Hamburg-Eppendorf

Indication

Radical cystectomy was performed in patients with muscle-invasive bladder cancer as well as in those with high-risk non–muscle-invasive disease refractory to intravesical instillation therapy. The choice of urinary diversion was individualized based on tumor stage and patient preference. Continent diversions were generally considered only in patients with organ-confined disease, whereas those with locally advanced tumors or significant comorbidities typically underwent ileal conduit or cutaneous ureterostomy.

Perioperative Workflow and Surgical Procedure

All patients underwent comprehensive preoperative evaluation including blood tests, electrocardiography, physical examination, abdominal ultrasound, and urinalysis. In patients with relevant cardiopulmonary comorbidities, additional assessments—such as pulmonary function testing, echocardiography, or myocardial perfusion imaging—were performed. Bowel preparation was administered one day before surgery using lavage solutions: 2–3 L polyethylene glycol with ascorbic acid (MOVIPREP®) in most cases, or magnesium sulfate (F.X. Passage® SL) in patients scheduled for ileal conduit diversion. Clinical staging was based on transurethral resection of the bladder tumor, and computed tomography (CT) scans of the abdomen and thorax. Bone scintigraphy was performed only in selected cases. A single preoperative dose of antibiotic prophylaxis (cefuroxime 1.5 g) was given. Postoperatively, metronidazole (500 mg twice daily) and cefuroxime (1.5 g three times daily) were continued

for three and five days, respectively. Pantoprazole 40 mg once daily was administered perioperatively to reduce gastric acid secretion and volume. Open radical cystectomy was performed using standard techniques, with a descending approach in females and an ascending approach in males. Pelvic lymph node dissection was routinely carried out following a limited template up to the iliac bifurcation, as defined in the randomized trial by Gschwend et al. [1]. Ureteroenteric anastomosis in patients undergoing ileal conduit diversion was performed using the refluxing Wallace technique [2]. In patients receiving a MAINZ pouch [3] or orthotopic neobladder, constructed as an ileal "S" bladder per Schreiter's technique [4], ureters were implanted refluxively. The previously used antirefluxive technique was abandoned due to increased rates of pyelonephritis and implantation stenosis.

Postoperative pain management included patient-controlled epidural or intravenous analgesia, supplemented with metamizole. Two Jackson-Pratt drains and bilateral ureteric splints were routinely placed. In patients with an ileal neobladder or MAINZ Pouch I, a transurethral Foley catheter was also inserted. Parenteral nutrition was administered until at least postoperative day 3 using NuTRIflex® Omega plus, Addel®, and Cernevit®. Neostigmine and metoclopramide were routinely given to promote bowel recovery and discontinued after the first bowel movement, at which point the nasogastric tube was removed. Oral intake of tea and water began on postoperative day 1, followed by regular food intake from day 3 onwards, adjusted individually based on recovery. Although no formal Enhanced Recovery After Surgery (ERAS) protocol was implemented, 17 of the 22 ERAS items proposed by Cerantola et al. [5], were routinely applied. Items 4, 9, 10, 15, and 22 were not fulfilled. In the absence of complications, ureteral splints were removed on postoperative days 10 and 11, followed by intravenous urography on day 12. In patients with a MAINZ pouch I or ileal neobladder, routine pouchography or cystography was performed before discharge.

REFERENCES

[1] Gschwend JE, Heck MM, Lehmann J, Rubben H, Albers P, Wolff JM, et al. Extended Versus Limited Lymph Node Dissection in Bladder Cancer Patients Undergoing Radical Cystectomy: Survival Results from a Prospective, Randomized Trial. Eur Urol. 2019;75:604-11.

[2] Wallace DM. Ureteric diversion using a conduit: a simplified technique. Br J Urol. 1966;38:522-7.

[3] Thuroff JW, Riedmiller H, Fisch M, Stein R, Hampel C, Hohenfellner R. Mainz pouch continent cutaneous diversion. BJU Int. 2010;106:1830-54.

[4] Schreiter F. [The S-bladder--a complete continent antireflux functional replacement of the bladder sphincter muscle function]. Urologe A. 1987;26:201-9.

[5] Cerantola Y, Valerio M, Persson B, Jichlinski P, Ljungqvist O, Hubner M, et al. Guidelines for perioperative care after radical cystectomy for bladder cancer: Enhanced Recovery After Surgery (ERAS®) society recommendations. Clin Nutr. 2013;32:879-87.

Suppl. Figure 1

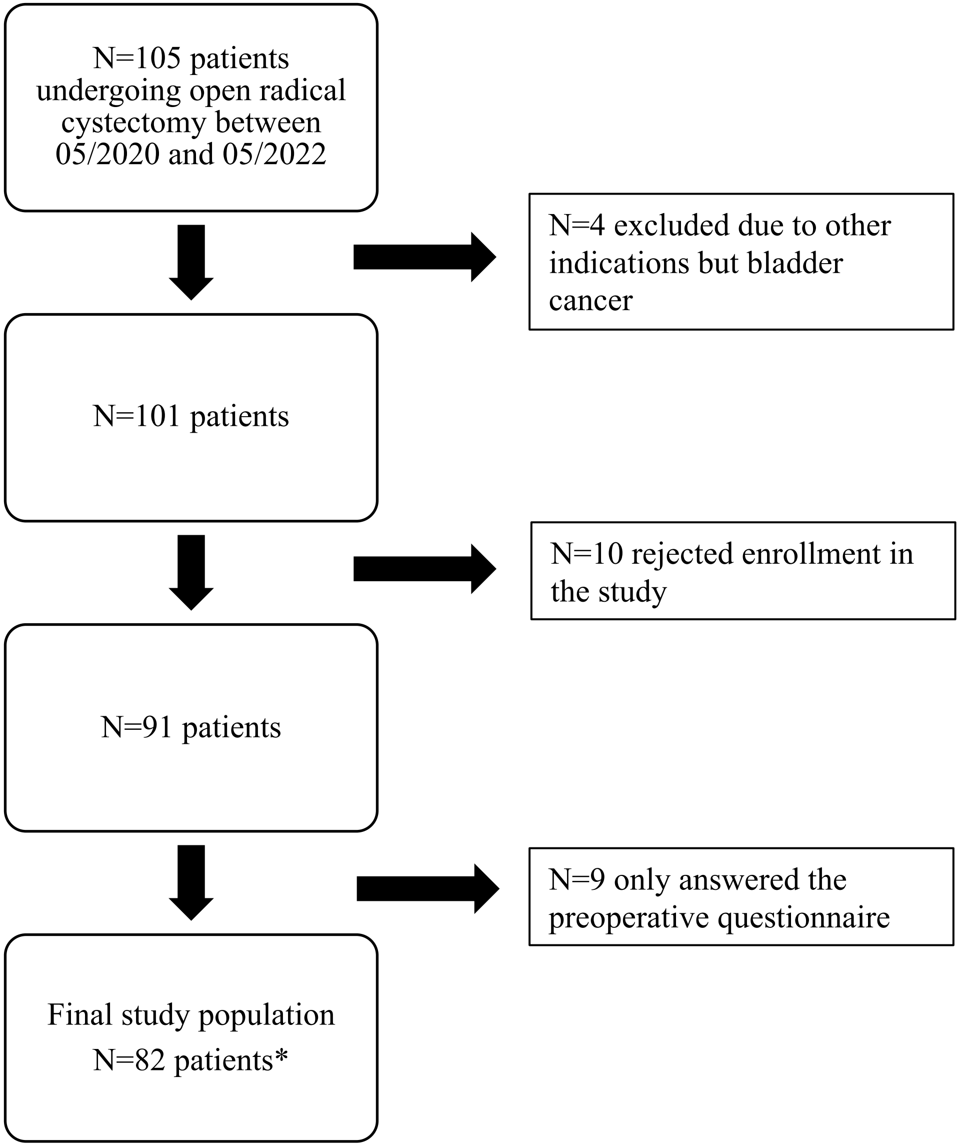

Suppl. Figure 2

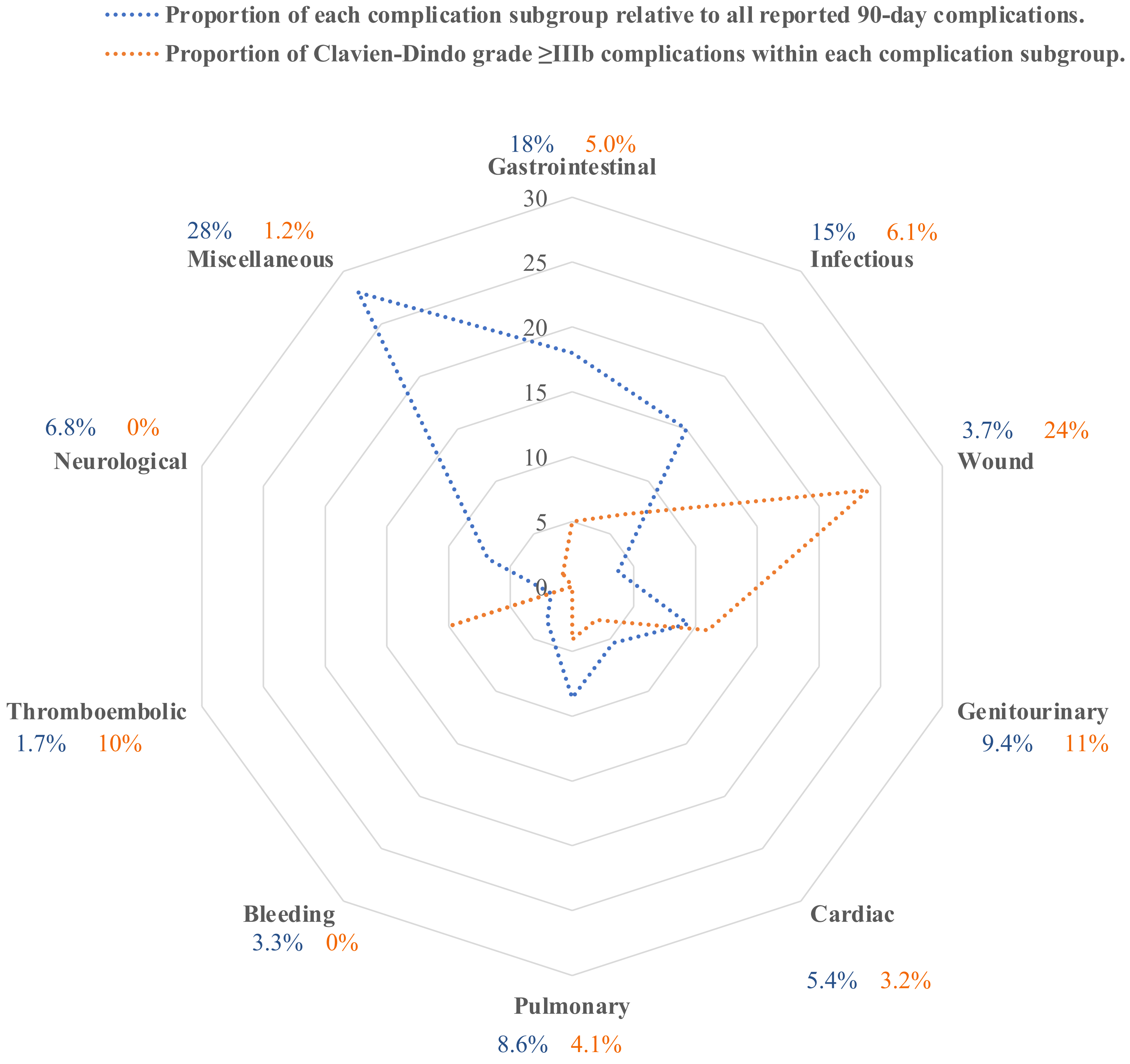

Supplement: Supplementary Data 2 [file mmc2.pdf]
